# Understanding functional abdominal pain disorders among children: a multidisciplinary expert consensus statement

**DOI:** 10.3389/fped.2025.1576698

**Published:** 2025-05-12

**Authors:** Yvan Vandenplas, Andy Darma, Flavia Indrio, Marion Aw, Mario C. Vieira, Boosba Vivatvakin, Suporn Treepongkaruna, Sylvia Cruchet, Bhaswati C. Acharyya, Rodrigo Vázquez, Chun Yan Yeung, Pedro Gutiérrez

**Affiliations:** ^1^ Department of KidZ Health Castle, UZ Brussel, Vrije Universitiet Brussels, Brussels, Belgium; ^2^ Department of Child Health, Dr. Soetomo General Academic Hospital, Surabaya, Indonesia; ^3^ Department of Experimental Medicine, University of Salento, Lecce, Italy; ^4^ Department of Paediatrics, National University Health System, Singapore, Singapore; ^5^ Centre for Paediatric Gastroenterology, Hospital Pequeno Príncipe, Curitiba, Brazil; ^6^ Department of Pediatrics, Chulalongkorn University, Thai Red Cross Society, Bangkok, Thailand; ^7^ Department of Pediatrics, Faculty of Medicine, Ramathibodi Hospital, Mahidol University, Bangkok, Thailand; ^8^ Human Nutrition Unit, INTA, University of Chile, Santiago, Chile; ^9^ Department of Paediatric Gastroenterology and Hepatology, Institute of Child Health, Manipal Hospital, Kolkata, India; ^10^ Department of Research, Hospital Infantil de México Federico Gómez, Mexico City, México; ^11^ Department of Gastroenterology and Hepatology, Hsinchu Municipal MacKay Children's Hospital, MacKay Medical College, Taipei, Taiwan; ^12^ Pediatric Research Division, Universidad Juarez del Estado de Durango, Durango, Mexico

**Keywords:** functional abdominal pain disorders, Rome criteria, microbiota–gut–brain interaction, abdominal pain, functional gastrointestinal disorder, probiotics

## Abstract

**Introduction:**

Functional abdominal pain disorders (FAPDs) are pediatric gastrointestinal conditions marked by chronic or recurrent abdominal pain without anatomical and/or biochemical abnormalities. This position paper guides primary care providers in the early diagnosis and management of FAPDs to improve the well-being of affected children and their families.

**Methods:**

A 12-member expert advisory board reviewed current approaches to diagnosing and managing FAPDs in children. Based on literature and discussions, 23 statements were drafted and voted on to achieve an acceptable level of agreement.

**Results:**

First-line healthcare professionals are key in diagnosing FAPDs, using ROME diagnostic criteria and recognizing red flags for accurate assessment and referrals. Comprehensive evaluation, including medical, dietary, and psychosocial history, physical exams, and basic tests helped to identify the initial triggers. Probiotics such as *Limosilactobacillus* (*L. reuteri*) DSM 17938 and *Lacticaseibacillus rhamnosus* (*L. rhamnosus*) help in alleviating functional abdominal pain (FAP) in children along with primary measures, such as dietary modifications [a balanced diet advocating moderation in fermentable oligosaccharides, disaccharides, monosaccharides, and polyols (FODMAP)-rich foods] and physical activity. Probiotics should be given for 6–8 weeks and can be resumed if symptoms recur. Cognitive-behavioral and hypnotic therapy also help, with remote options such as web-based, compact disk (CD)-based or application-based tools available.

**Discussion:**

This position paper provides expert insights to guide primary care providers in diagnosing and managing FAPDs, equipping them to make informed decisions for effective management of FAPDs.

## 1 Introduction

Functional abdominal pain disorders (FAPDs) are part of a large group of gastrointestinal (GI) disorders characterized by recurrent abdominal pain that cannot be fully explained by another medical condition (1). FAPDs affect infants and children worldwide, impacting approximately 13.5% [95% confidence interval (CI): 11.8–15.3] of the pediatric population. The prevalence rates were reported to be higher in South America (16.8%) and Asia (16.5%) compared to Europe (10.5%) (2). Recurrent abdominal pain is one of the most frequent reasons for pediatric consultations. About 90% of children with recurrent abdominal pain are diagnosed with FAPDs, with only 10% of cases revealing an identifiable somatic cause (3). These chronic disorders diminish the quality of life (QoL) of the affected children and their families and elevate the likelihood of anxiety, depression, school absenteeism, and a decline in academic performance due to recurrent episodes (4, 5). FAPDs are classified into distinct entities based on the Rome IV criteria. Within this categorization, notable subtypes include irritable bowel syndrome (IBS), functional dyspepsia, abdominal migraine, and functional abdominal pain–not otherwise specified (FAP-NOS). In the new Rome IV criteria, the frequency of pain symptoms was revised to at least four times per month for at least 2 months to fulfil the criteria for diagnosis (1).

Despite the well-established criteria for FAPDs outlined by the ROME IV committee, these conditions are frequently not well comprehended. Diagnosis presents a challenge due to its multifaceted origins and the presence of overlapping disorders (1, 6). Although these disorders have historically been described as “functional,” they are now considered as “disorders of gut–brain interaction” (DGBI), emphasizing that the term “functional” should not be misconstrued to imply a nonorganic condition (6). Moreover, the ROME IV committee highlights the importance of diagnosing FAPDs only after an appropriate evaluation, particularly when the symptoms cannot be fully explained by another medical condition (1).

DGBI can lead to substantial financial burdens on both families and healthcare systems (7). Insufficient awareness and understanding of FAPDs often lead to delayed or inadequate interventions. The varied presentation of symptoms and overlapping of other GI disorders further complicates timely diagnosis (6). This highlights the necessity to devise practical solutions for empowering healthcare professionals (HCPs) in promptly identifying FAPDs.

## 2 Methodology

### 2.1 Expert selection process

The advisory board panels comprised experts specializing in pediatric gastroenterology from various countries, carefully selected to ensure a diverse and comprehensive representation of knowledge, clinical experience, and regional treatment practices. The selection process aimed to include experts with relevant contributions to research and clinical management of FAPDs, ensuring a well-rounded perspective on current challenges and emerging strategies. Particular emphasis was placed on geographic diversity to incorporate varying healthcare systems, diagnostic approaches, and treatment methodologies. This inclusive selection aimed to bridge gaps in knowledge, promote international collaboration, and provide practical guidance to primary care providers for timely diagnosis and early intervention in children with FAPDs.

### 2.2 Evidence review

The primary goal of the expert committee meeting was to facilitate thorough discussion and formulate expert statements regarding:

• Practical strategies to assist primary care providers in the recognition and prompt diagnosis of FAPDs for early treatment interventions
• Understanding the role and effectiveness of various treatment strategies in managing FAPDs

To support these discussions, an extensive literature review was conducted to source relevant articles from reputable databases such as PubMed, Google Scholar, and the Cochrane Library. The review encompassed articles published between March 2000 and June 2023. The search strategy employed relevant free-text keywords combined with appropriate Boolean operators (AND, OR). Some of the keywords used in the search were “Functional abdominal pain,” “Abdominal pain,” “Functional abdominal pain disorders,” “Functional gastrointestinal disorders,” “irritable bowel syndrome,” “Functional dyspepsia,” “Abdominal migraine,” “Management,” “Guidelines,” and “Probiotics.”

### 2.3 Consensus development and voting process

**Figure 1:**
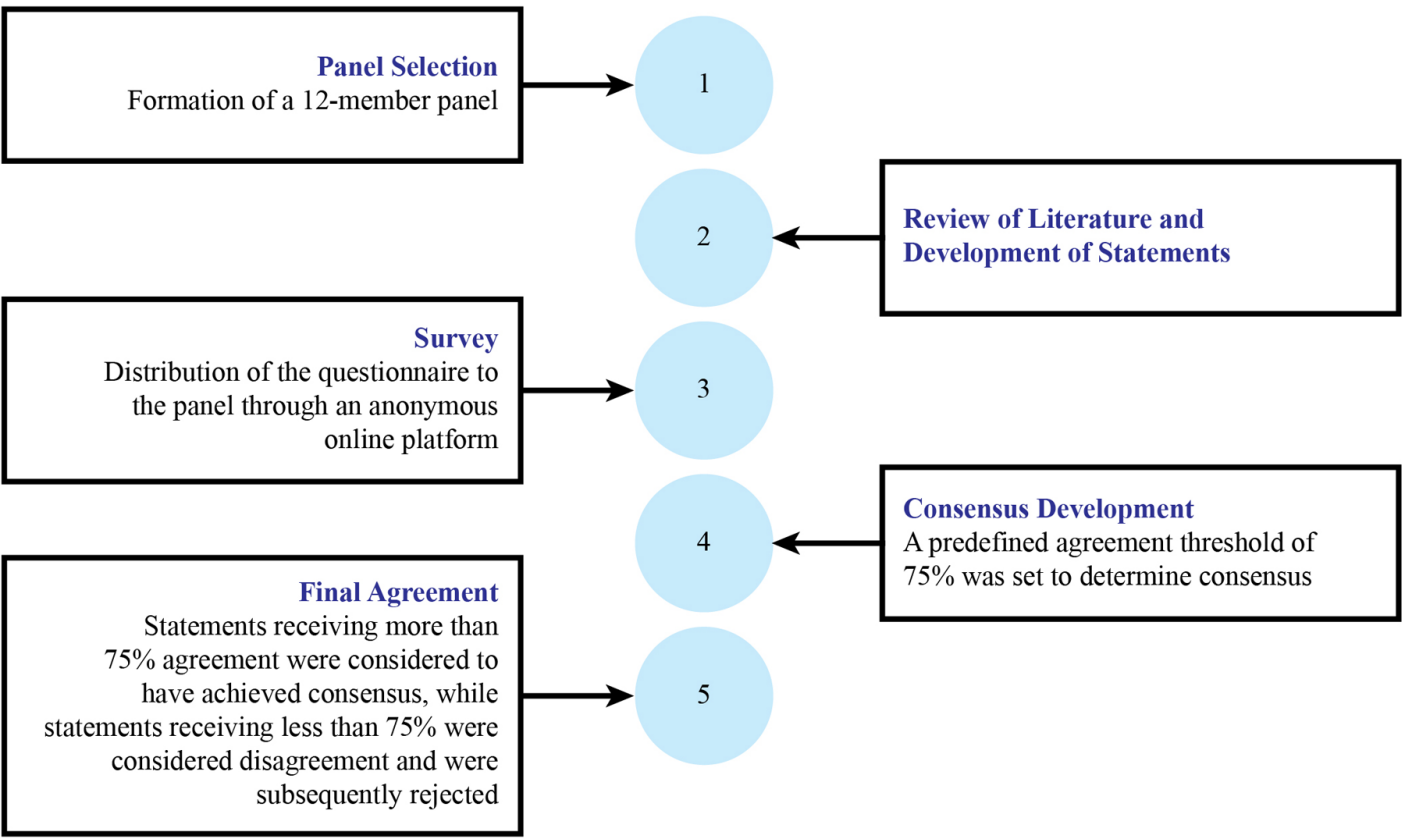
Overview of the consensus process used to create and achieve the consensus statement.

To ensure a structured and transparent formulation of expert recommendations, the committee followed a rigorous consensus-building process (Figure 1). After in-depth discussions during meetings, provisional expert statements were developed based on the collective insights of the panel. These statements were further refined by designated subgroup members after the meeting to ensure clarity, clinical relevance, and alignment with current evidence.

Once finalized, the refined statements were circulated among all expert panel members through an anonymous online voting system to eliminate bias and encourage independent judgment. Each participant had the option to either agree or disagree with the statements. A predefined agreement threshold of 75% (≥9/12 authors) was set to determine consensus—statements receiving less than 75% agreement were considered to have significant disagreement and were subsequently rejected. This rigorous methodology ensured that the final expert recommendations reflected a high level of agreement, clinical applicability, and credibility for guiding primary care providers in managing FAPDs in children.

## 3 Results

A survey was conducted through online voting on the 23 finalized statements by the expert group. A total of 12 experts (*n* = 12) participated in the survey. After receiving the responses from all the participants, the survey results were analyzed. The agreement on the statements ranged from a high agreement score (100%), where all experts agreed with the statements, to a low score (58%), indicating significant disagreement. Based on the survey results, all the statements on the diagnosis and management of FAPDs achieved the established consensus agreement criteria to qualify (outlined in Tables 1, 2). However, one statement that suggested considering constipation during the diagnostic process without causing any undue alarm did not qualify for the consensus score (58%) and was eliminated from the statements.

**Table 1:** Experts' statements on screening and diagnosis of FAPDs in primary care.

Sr. no.	Statement	Agreement (%) (*n* = 12)
1.	GPs have a vital role in the initial assessment of FAPDs. Familiarity with the ROME IV diagnostic criteria and potential red flags associated with FAPDs can facilitate early diagnosis and referrals to specialist care only when necessary.	100%
2.	A comprehensive clinical assessment, including detailed medical and diet history, along with a physical examination, should be performed in children presenting with FAPDs.	100%
3.	Assessing the psychosocial history of both the child and parents may offer insights into stress-related triggers.	100%
4.	The higher the number of alarm symptoms present, the higher the likelihood of an organic disease.	100%
5.	GPs should prioritize noninvasive tests, such as stool examination and stool occult blood tests, over invasive procedures, such as endoscopy or colonoscopy, during the initial diagnostic evaluation.	100%
6.	Endoscopy should be used to examine and assess abnormalities caused by *H. Pylori* infection, not solely to identify the presence of *H. pylori.*	83%
7.	Dietary history provides important clues for diagnosis. Employing a specific history-taking approach known as “functional abdominal pain-specific dietary history” is crucial for screening lactose intolerance in children.	75%

FAPD, Functional abdominal pain disorder; GP, General physician; *H. pylori, Helicobacter pylori*; *n*, total number of experts who participated in the survey.

**Table 2:** Experts' statements on treatment approaches of FAPDs.

Sr. no.	Statement	Agreement (%) (*n* = 12)
1.	It is advisable to follow a balanced diet that stresses moderation in foods rich in FODMAP.	83%
2.	The low-FODMAP diet helps alleviate digestive issues caused by excessive FODMAP consumption. By following this diet, children may naturally decrease their intake of junk food and sweets, further aiding symptom relief.	92%
3.	Probiotics can be considered along with dietary modifications and physical activity.	100%
4.	During probiotic treatment, monitor symptoms for a few weeks. If successful, continue; if no response is observed from the treatment, reassess the diagnosis or modify the treatment.	75%
5.	Regardless of the type of probiotic chosen, it is recommended to use it for 6–8 weeks.	92%
6.	Evaluating the efficacy of probiotics in clinical practice after 2–3 months may be impractical, as patients may discontinue the treatment if they notice no improvement within the initial 2–3 weeks.	92%
7.	*L. reuteri* DSM 17938 alleviates FAP in children by reducing both the frequency and severity of pain episodes.	92%
8.	Probiotics such as *L. rhamnosus* and *L. reuteri* DSM 17938 for FAPDs have been shown to be beneficial, with *L. reuteri* potentially being more suitable for managing FAP-NOS.	100%
9.	Pharmacological management of FAPDs should entail selecting the specific drug class based on the patient subtype.	92%
10.	Psychotherapy such as CBT and hypnotic therapy may aid children, providing support and alleviating pain without causing harm.	100%
11.	Exploring remote treatment options for hypnotherapy, including web-based, CD-based, and application-based guidance sessions is recommended.	92%
12.	Follow-up in patients with FAPDs should be consistent to provide reassurance and support, which are crucial components of patient management. This could help patients and parents feel more at ease and confident about the diagnosis.	100%
13.	A practical strategy for the attending physician is to implement “frontline CBT” to alleviate symptoms. This involves teaching the patient techniques to divert attention from the abdominal pain or to “cheat the brain,” by engaging in activities such as dancing, playing sports, making music, or singing.	100%
14.	FAPDs entail gut–brain interaction rather than being a severe illness. Most children with FAPDs often develop additional disorders related to gut–brain interaction over time.	92%
15.	It is crucial to highlight the importance of adopting a healthy lifestyle and offer reassurance to both parents and children.	100%

CBT, cognitive behavioral therapy; FAP, functional abdominal pain; FAPD, functional abdominal pain disorder; FAP-NOS, functional abdominal pain–not otherwise specified; FODMAP, fermentable oligosaccharides, disaccharides, monosaccharides, and polyols; *L. reuteri*, *Limosilactobacillus reuteri*; *L. rhamnosus*, *Lacticaseibacillus rhamnosus*; *n*, total number of experts who participated in the survey.

### 3.1 Statements that did not achieve consensus

Based on the survey responses, 22 statements on the diagnosis and management of FAPDs achieved the set consensus agreement criteria for the statement to qualify. The majority of experts agreed that constipation is a common symptom in both functional constipation and irritable bowel syndrome with constipation (IBS-C), often occurring without indicating a serious underlying condition. Consequently, one statement regarding the consideration of constipation during the diagnostic process without causing undue alarm did not reach the consensus score (58%) and was eliminated (Table 3).

**Table 3:** Statement that did not achieve consensus.

Sr. no.	Statement that did not qualify for the agreement score	Agreement (%)
1.	Constipation should be considered in the diagnostic process without causing undue alarm.	58%

## 4 Discussion

### 4.1 Pathophysiology and risk factors

FAPDs encompass complex and multifaceted interactions which can be explained by a comprehensive biopsychosocial model. It involves heightened visceral hypersensitivity and central hypervigilance, possibly due to genetic predisposition, early-life events, and sensitizing psychosocial and medical factors, combined with disordered microbiota–gut–brain interaction, which represents the bidirectional communication pathway between the gut and brain via the gut microbiota. In this context, gut dysbiosis has also been demonstrated in patients with functional gastrointestinal disorders (FGIDs) (6).

#### 4.1.1 Visceral hypersensitivity, central hypervigilance, and microbiota–gut–brain interaction

**Figure 2:**
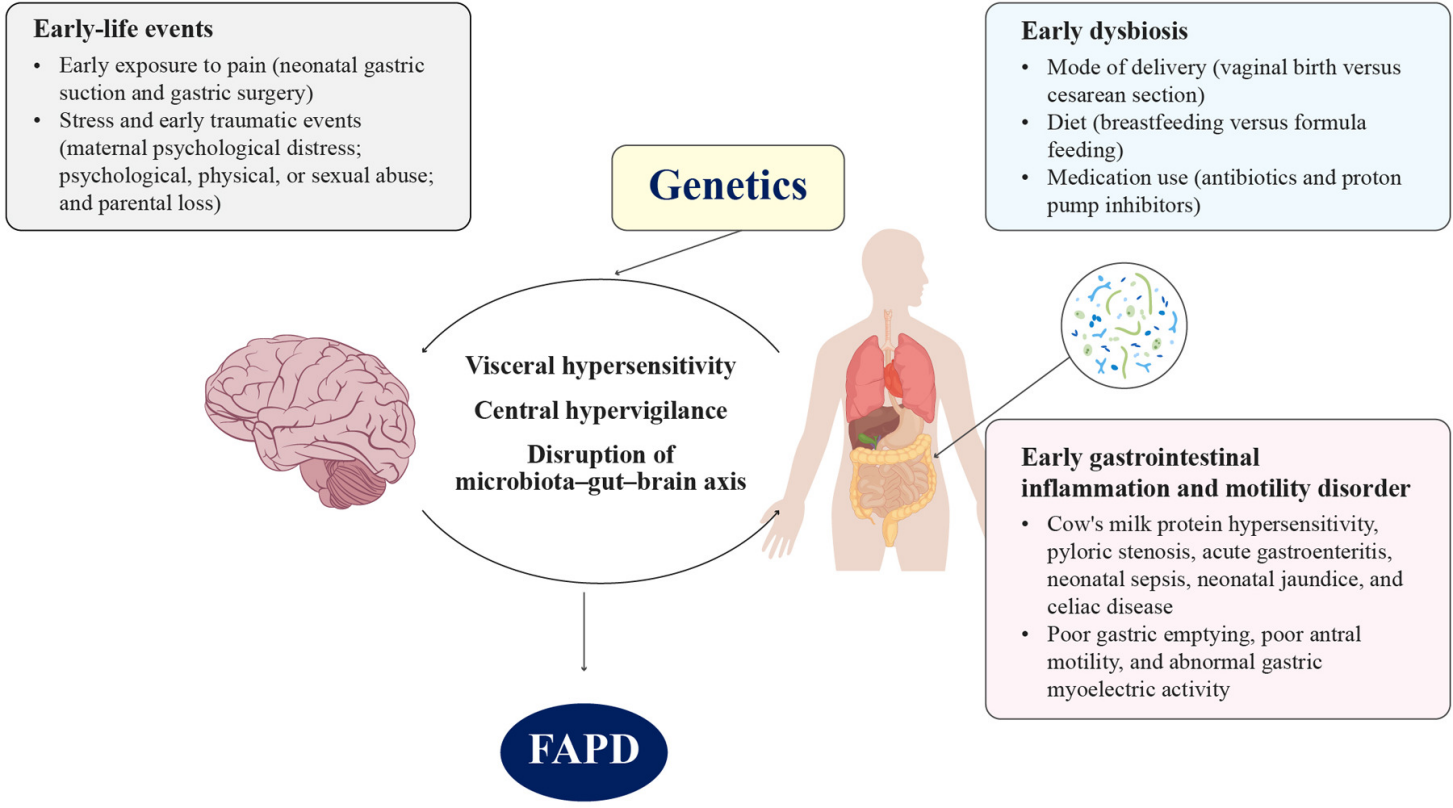
Role of early-life events including early dysbiosis, early gastrointestinal (GI) inflammation, and motility disorder in the pathophysiology of FAPDs (11, 14, 15). FAPD, functional abdominal pain disorder.

Enhanced perception of visceral stimuli due to increased sensitivity of visceral afferent pathways (visceral hypersensitivity) or central amplification of visceral input is one of the concepts that has persistently been implicated in the pathophysiology of FGIDs in children (8). Visceral hypersensitivity can be caused by aberrations in the visceral nociceptive neuraxis, ion channels, neurotransmitter receptors, trophic factors, and central pain processing (9). Compared with their control counterparts, children with visceral hypersensitivity often experience a reduced sensory threshold for pain (10). Visceral hypersensitivity is also linked to the descending modulation of visceral nociceptive pathways by the autonomic nervous system, hypothalamus–pituitary–adrenal axis, and certain psychological factors (9). Studies have shown that stress, anxiety, and depressive disorders are some of the common factors associated with FAPDs (2, 4, 8). One study found that mood disorders preceded FGIDs in one-third of children, while in two-thirds, FGIDs preceded the mood disorder, indicating primary gut mechanisms as the drivers of FGIDs (10). Central hypervigilance simply represents the altered processing of “pain” sensations received by the brain from the sensory fibers of the gut (11). Studies have demonstrated an association between early painful experiences in neonates and children and the occurrence of visceral hypersensitivity, hypervigilance, and FAPDs (12–14). Apart from pain exposure, stress, early traumatic events, and GI inflammation/disorders are some of the other early-life events implicated in the pathophysiology of FAPDs (15). Both visceral hypersensitivity and central hypervigilance are caused by insults to the gut–brain–microbiota interaction and neuroimmune interactions within the gut (11). Factors that disrupt or alter the gut microbiota can disrupt the integrity of the enteric nervous system, leading to hypersensitivity in the GI tract and hypervigilance in the brain (6). This awareness about the complex interaction between these factors has improved the understanding of FAPD pathogenesis in children, including the concept of “early-life programming” (11). Figure 2 illustrates the role of early-life events, including dysbiosis, GI inflammation, and motility disorders, along with genetic predisposition, in the pathophysiology of FAPDs in children (11, 14, 15).

#### 4.1.2 Risk factors

Age, sex, psychosocial factors, and genetic factors play a significant role in the etiology of FAPDs. A meta-analysis indicated a higher prevalence of FAPDs among girls than boys {15.9% vs. 11.5%, pooled odds ratio [OR]: 1.5} (2). These results align with findings from adolescent studies, where multiple logistic regression analysis showed a significant association between FAPDs and female sex {OR: 3.3, 95% confidence interval [CI]: 1.7–6.4} (16). Female children have been found to exhibit significantly higher levels of trait anxiety and somatization [*p* = 0.04 and *p* = 0.005, respectively] (17). The association between age and the prevalence of functional abdominal pain (FAP) was investigated in 36 studies in a meta-analysis. No significant difference was observed in the prevalence of FAPDs between children younger than 12 years and those aged 12 years [12.4% vs. 13.8%, pooled OR: 0.9, 95% CI: 0.5–1.4, *p* = 0.62] (2). A Chinese cross-sectional survey involving 2,344 children aged 6–17 years found no significant difference in the prevalence of FAPDs among the following age groups: 6–9 years, 10–13 years, and 14–17 years [*p* = 0.488] (5). In the same study, academic stress, academic performance below parental expectations, strained relationships with parents, and sleep disorders (difficulty falling asleep or waking up early) were independent risk factors for FAPDs in children (5). Several studies have reported the association of FAPDs with multiple psychosocial factors such as anxiety, depression, emotional sensitivity, somatization, and lower coping efficacy (2, 17–19). Early-life events seem to play an important role in the occurrence of FAPDs (see Figure 2). Bonilla et al. underscored the significance of early-life events, noting that early childhood represents a crucial stage during which psychological or physical trauma can trigger visceral hyperalgesia/hypersensitivity. They further suggested that timely intervention during this period could play a critical role in preventing these chronic debilitating conditions (15).

### 4.2 Screening and diagnosis

**Figure 3:**
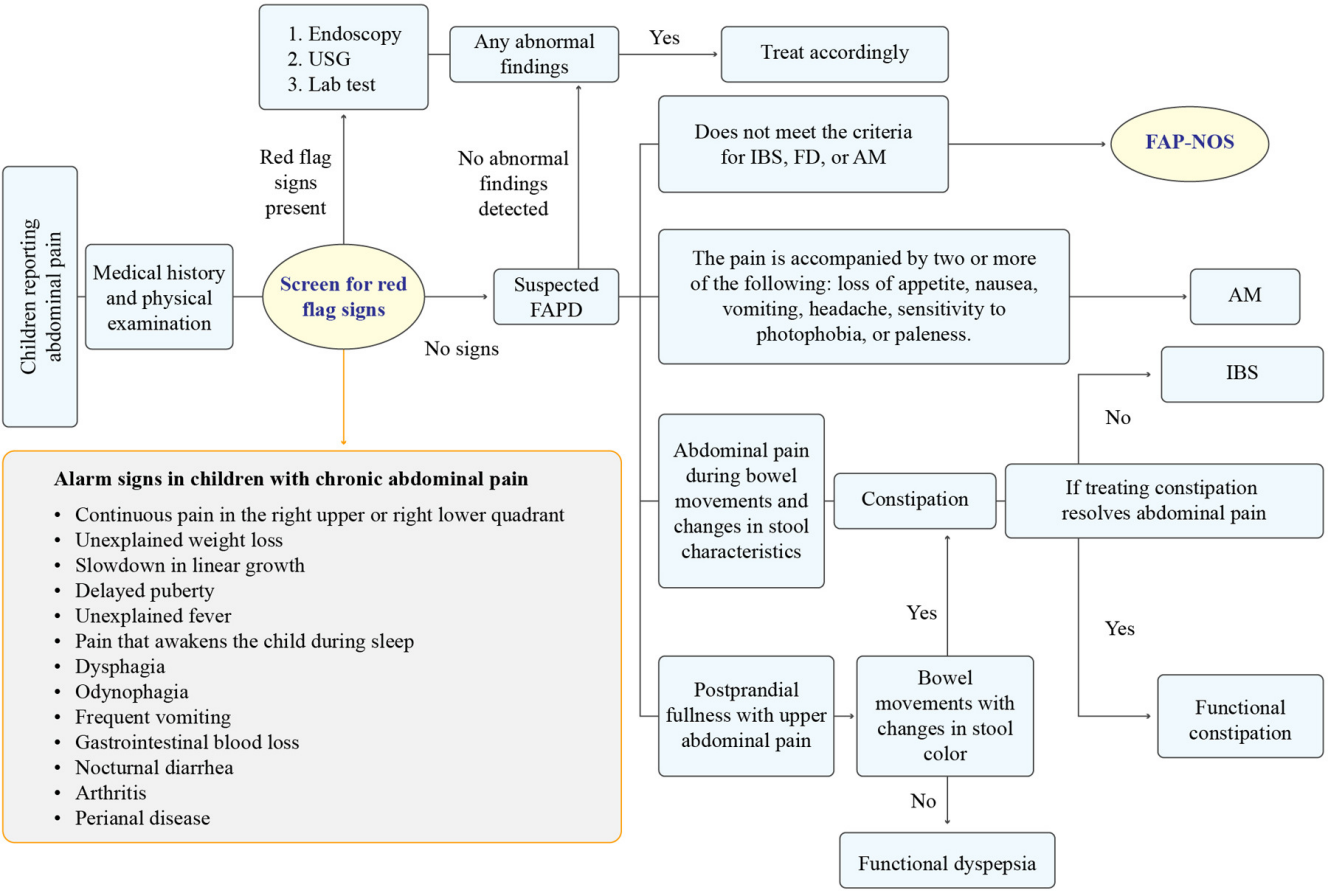
Stepwise approach to diagnose FAPDs in children (1, 24). AM, abdominal migraine; FAPD, functional abdominal pain disorder; FAP-NOS, functional abdominal pain–not otherwise specified; FD, functional dyspepsia; IBS, irritable bowel syndrome; USG, ultrasonography.

The diagnosis of FAPDs in children involves a comprehensive approach that considers both physical and psychosocial factors. Collaboration between healthcare professionals, thorough assessments, and adherence to established diagnostic criteria contribute to an accurate diagnosis and appropriate management of the condition. Obtaining a comprehensive patient history and conducting a meticulous physical examination are the essential steps for confirming the diagnosis of FAPDs and assuring patients of the benign nature of the condition. Medical history should also include exploring the possibility of abuse, given its potential link with FAPDs (20). Apart from general examinations, physical examinations may include perianal and rectal examinations to identify perianal pathology but should be reserved for those in whom an organic pathology is suspected (21). Patients typically present with potential alarm symptoms/signs. Figure 3 outlines the diagnostic algorithm for FAPDs in children. It begins with a medical history and physical examination, followed by screening for red flag signs (e.g., unexplained weight loss, delayed puberty, and GI blood loss). The presence of red flags warrants further investigations, including endoscopy, ultrasound (USG), and laboratory tests. If no red flags or abnormal findings are detected, FAPD is suspected. It is crucial to identify these alarming signs to rule out FAPDs (6). FAP-NOS encompasses cases of episodic or constant abdominal pain that does not exclusively occur during normal physiological events and does not fulfil the criteria for other FAPDs. Research, particularly on FAP-NOS in children, especially those with concurrent IBS, has suggested that children with FAP-NOS usually do not display heightened rectal sensitivity. Additionally, studies have indicated that these children exhibit low antral contractions and experience delayed rates of gastric emptying (20, 22, 23).

#### 4.2.1 Challenges in the diagnosis of FAPDs in primary care

According to experts, HCPs face significant challenges in screening and diagnosing FAPDs due to several factors, including limited evidence guiding the diagnostic criteria and treatment strategies, a lack of awareness among HCPs about the condition, and difficulty in understanding the ROME IV diagnostic criteria. Experts have highlighted another significant challenge in discerning between “ruling in” or “ruling out” the diagnosis of FAPDs, which relies more on clinical judgment rather than specific diagnostic tests, making it challenging to definitively establish the presence of the condition.

The Rome IV criteria, established in 2016, serve as the prevalent diagnostic standard for confirming FAPDs. When a child meets specific clinical criteria outlined in the Rome IV, a FAPD diagnosis can be established without the necessity for further testing. Figure 3 outlines the diagnostic algorithm for FAPDs as suggested by the expert panel (1, 24).

In instances where an organic disorder is suspected, clinicians must carefully select appropriate diagnostic tests. These may encompass a range of laboratory assessments, including initial tests such as complete blood count, serological testing for conditions such as celiac disease, and evaluation of fecal calprotectin levels. Advanced diagnostic tests, such as imaging studies (abdominal ultrasound) and, in certain cases, endoscopy with biopsies for histological examination, are also employed. These tests serve as screening tools for underlying conditions with subtle “alarm findings” that might be missed in the initial diagnosis. In the absence of evident organic pathology, clinicians should consider the possibility of a FAPD diagnosis. This involves assessing whether the patient's symptom profile aligns with the criteria for any specific FAPD, such as functional dyspepsia, IBS, abdominal migraine, or FAP-NOS, as defined by the Rome IV criteria (Supplementary Table S1) (1, 6, 11).

### 4.3 Treating approaches

Managing FAPDs can be complex due to their multifaceted nature. Typically, treatment involves a multidisciplinary approach that incorporates pharmacological and nonpharmacological therapies and psychological interventions customized to meet the specific needs of each patient. Table 4 elucidates different approaches for the management/treatment of FAPDs (6, 25).

**Table 4:** Treatment approaches of FAPDs (6, 25).

Approach type	Treatment components
Nonpharmacological approaches	A. Dietary changes • Low-FODMAP diet • Gluten-free diet • Prebiotics/probiotics B. Patient education C. Planning further treatment after validation of symptoms
Pharmacological approaches	A. Laxatives B. Analgesics C. Antibiotics D. Antispasmodics E. Tricyclic antidepressants F. Serotonin–norepinephrine reuptake inhibitors G. Progressive therapy options: • Gabapentin • Pregabalin
Psychological approaches	A. Cognitive behavioral therapy B. Hypnotherapy C. Biofeedback therapy

FAPD, functional abdominal pain disorder; FODMAP, fermentable oligosaccharides, disaccharides, monosaccharides, and polyols.

#### 4.3.1 Nonpharmacological approaches for the management of FAPD

##### 4.3.1.1 Dietary modifications

While dietary factors are considered to play a role in the development of FAPDs, the advantages of dietary modifications are still a subject of controversy. With the growing recognition of the importance of gut microbiota, there is an increasing focus on interventions designed to influence gut microbiota for the management of FAPDs. For children diagnosed with FAPDs, dietary interventions such as the implementation of a low-fermentable oligosaccharides, disaccharides, monosaccharides, and polyols (FODMAP) diet; prebiotics; and probiotics can be considered (23, 26). A recent systematic review and meta-analysis reviewed the efficacy and safety of fibers, FODMAP diet, fructans, fructose-restricted diet, prebiotic (inulin), serum-derived bovine immunoglobulin, and vitamin D supplementation for pediatric FAPD. The study concluded that the use of fiber can be considered on daily basis (27)*.* Although dietary modification to address specific aspects of IBS is potentially promising, the effectiveness of such interventions for children with FAP-NOS remains uncertain, primarily due to lack of substantial evidence (6).

##### 4.3.1.2 Effectiveness of FODMAP diet for FAPD management

Some studies have indicated that the FODMAP diet can influence the gut microbiome and heighten visceral nociception by inducing dysbiosis. The rationale for using a low-FODMAP diet is based on the premise that reducing the intake of short-chain fermentable carbohydrates may help prevent their osmotic effect, thereby lowering water volume in the small intestine. Additionally, it limits the excessive fermentation of FODMAPs by colonic microbiota, reducing gas production and potentially alleviating recurrent abdominal pain (28). Implementing restrictive diets such as the low-FODMAP diet should be done under the supervision of a clinician due to the potential risk of nutritional inadequacy and the development of unhealthy eating behaviors (6, 26). The complexities surrounding microbiome analysis of stool samples to determine which subset of patients benefit from a low-FODMAP diet is still an unresolved issue as it requires standardized criteria for stool sampling and storage (6, 21). It is important to acknowledge that further studies are needed to deepen our understanding of the long-term efficacy, potential side effects, and applicability of a low-FODMAP diet across diverse populations as a recent systematic review reported insufficient evidence for or against the efficacy and safety of using a low-FODMAP diet for the management of children with FAPD (29)*.* Similarly, a randomized controlled trial comparing the low-FODMAP diet to a standard diet found no statistically significant differences in abdominal pain intensity or stool consistency in children with FAPD. The control group which followed NICE guidelines, showed greater improvement in symptoms (30)*.* According to experts, there is a common misconception surrounding the FODMAP diet, often misunderstood as advocating for a completely FODMAP-free diet, particularly for FAP-NOS. However, implementing a FODMAP-free diet is challenging in practical terms. Critics argue that overly restrictive FODMAP diet may lead to nutritional deficiencies or eating disorders in children. These concerns are particularly relevant in the pediatric populations, where dietary adequacy and long-term adherence are critical considerations (31, 32).

##### 4.3.1.3 Probiotics

Probiotics are live microorganisms that, when given in sufficient quantities, provide a positive health effect to the host (33). Numerous studies have explored the effectiveness of probiotics utilizing various organisms, such as *Lacticaseibacillus rhamnosus GG* (LGG), *Lactobacillus acidophilus*, *Lacticaseibacillus paracasei, and Lactiplantibacillus plantarum* DSM 9843, for treating FAPDs, with a focus on adults with IBS. While some combinations or specific species show promise in managing FAPDs, their role remains uncertain due to study limitations, such as sample size, blinding, variations in probiotic types, and dosing (34).

A randomized controlled trial showed that *L. reuteri DSM* 17938 could potentially alleviate symptoms and enhance the overall QoL for individuals dealing with functional abdominal pain (35). In another systematic review and meta-analysis of randomized controlled trials up to 1 April 2020, examining probiotic interventions for functional abdominal pain in children, nine trials (702 children, 506 with functional abdominal pain, aged 4–18 years) were included. The analysis, involving eight studies with a total of 641 children, focused on two probiotic strains: *LGG and L. reuteri* DSM 17938. The results showed a significant reduction in pain intensity (6 trials, *n* = 380, mean difference: −1.24, 95% CI: −2.35 to −0.13) and an increase in the number of days without pain (2 trials, *n* = 101, mean difference: 26.42, 95% CI: 22.67–30.17) in children taking *L. reuteri* DSM 17938. On the other hand, LGG supplementation did not yield any significant benefits in treating FAP (1 trial out of 3 studies, *n* = 47, RR: 2.88, 95% CI: 0.64–12.82, random-effects model). This research highlights the effectiveness of *L. reuteri* in decreasing pain intensity in children with functional abdominal pain (36).

In another randomized controlled trial involving children aged 4–18 years diagnosed with FAP or IBS, participants were randomly assigned to receive either *L. reuteri* DSM 17938 at a dosage of 10⁸ colony-forming units (CFUs) daily or a placebo. The findings suggested that administering *L. reuteri* DSM 17938 potentially lead to a decrease in pain intensity and significantly increased the number of pain-free days in children diagnosed with FAP and IBS (37).

In a randomized controlled trial comparing polymicrobial probiotic to mono-strain probiotic, the polymicrobial probiotic group reported more children without pain, while the overall pain scores did not significantly differ from the mono-strain group (38).

##### Effectiveness of probiotics in the management of FAPDs

According to the guidelines established by the European Society for Paediatric Gastroenterology, Hepatology and Nutrition (ESPGHAN) in 2022, *L. reuteri* DSM 17938, administered at a daily dose ranging from 10^8^ CFU to 2 × 10^8^ CFU reduces the intensity of pain in children diagnosed with FAP (39). According to the World Gastroenterology Organisation Global Guidelines, for children diagnosed with FAP-NOS, certain probiotic strains such as *L. reuteri* DSM 17938 have shown the capability to relieve abdominal pain and improved the overall QoL in children experiencing FAP (40). Although, the evidence from different published studies underscores the potential of supplementing with *L. reuteri* DSM 17938 as a promising therapeutic strategy for FAPDs (Table 5); many studies lack robust placebo controls making it difficult to ascertain the true efficacy of probiotics (38).

**Table 5:** Evidence on the use of *L. reuteri* DSM 17938 for FAPD (27, 29–31, 34–36).

Study	Participants; duration; location; age group	Key results
Trivic et al. (36) (Meta-analysis)	9 studies included (*n* = 702); 4–16 years	Significantly reduced pain intensity and increased the number of days without pain (*p* = 0.21)
Rahmani et al. (33)	125; 4 weeks; India; 6–16 years	Significantly decreased the frequency, severity, and duration of abdominal pain; improved the pain pattern (*p* < 0.001)
Jadresin et al. (37)	46; 3 months + 1 month follow-up; Croatia; 4–18 years	Significantly increased days without pain and reduced intensity of pain at 4 months (*p* < 0.05)
Maragkoudaki et al. (41)	54; 4 weeks + 4 weeks follow-up; Greece, Slovenia, and Poland; 5–16 years	Significantly decreased child school absenteeism as well as the use of drugs to relieve pain (*p* = 0.72)
Weizmann et al. (35)	101; 4 weeks + 4 weeks follow-up; Israel; 6–15 years	Significantly reduced the frequency (*p* < 0.01) and intensity of pain (*p* < 0.02)
Chumpitazi et al. (42)	8; 7 days; Texas; 7–16 years	Significantly reduced the pain frequency, pain severity, and pain-related interference with activities (*p* < 0.05)
Romano et al. (43)	56; 4 weeks suppl. + 4 weeks follow-up; Italy; 6–16 years	Significantly reduced the severity of abdominal pain during *L. reuteri* DSM 17938 intake (*p* < 0.001)

*L. reuteri*, *Limosilactobacillus reuteri*.

Experts have highlighted a significant knowledge gap among HCPs regarding the use of probiotics in clinical settings for managing FAPDs. Probiotic interventions alleviate symptoms associated with FAPDs by restoring a balanced gut microbiota.

#### 4.3.2 Pharmacological approaches for managing FAPDs

When pharmacological interventions are considered suitable, the management of functional abdominal pain often includes some commonly used drugs such as laxatives, analgesics, antispasmodics, tricyclic antidepressants (TCAs), and serotonin–norepinephrine reuptake inhibitors (SNRIs). Based on the published study, a few children reported finding relief by taking antispasmodic medications, such as hyoscyamine or dicyclomine (22, 44). Antispasmodics are recommended as the first line of treatment for FAPDs. These medications may be used as a continuous maintenance treatment or to alleviate acute symptom episodes, depending on the symptoms (6, 22, 44). Peppermint oil or menthol has demonstrated efficacy in alleviating FAP through its antispasmodic mechanism. However, they should not be used in children under the age of 2 years due to their potential respiratory compromised effect.

The effectiveness of low daily doses of antidepressants, especially TCAs, has been demonstrated in addressing chronic pain and relieving symptoms across various painful FGIDs, including IBS (6, 23, 44, 45). In addition, the use of prokinetic drugs is recommended for the management of functional dyspepsia (6). There is currently inadequate evidence to substantiate the effectiveness of pharmacological treatments in children with FAPDs (23, 44, 45). Also, pharmacological therapy for the management of FAPDs has yielded unsatisfactory results. Future research endeavors should prioritize investigating the factors influencing the extent of placebo effects, aiming to discern ways to mitigate their impact in drug trials or leverage them effectively during therapeutic interventions. According to experts, the pharmacological management algorithm should undergo revision to incorporate a dedicated section for abdominal pain, recognizing its importance in patient care. Enhancing the algorithm would enable HCPs to cater to the diverse needs of patients, including those requiring specific drugs.

#### 4.3.3 Psychotherapy for the management of FAPDs

Research has established the substantial impact of psychological factors on treatment outcomes once symptoms of FAPDs are evident. In children with FAPDs, the presence of anxiety, depression, somatization (expressing multiple physical symptoms), and catastrophizing is associated with increased symptom severity, greater impairment in daily functioning, and prolonged persistence of the condition. Clinical data, supported by evidence from trials, highlight the effectiveness of psychological interventions, such as cognitive behavioral therapy and hypnotic therapy, in managing FAPDs. These interventions have proven to be valuable in reducing symptoms, mitigating disability, and enhancing the overall QoL in children dealing with FAPDs (22, 23). Experts have emphasized that FAPDs are not serious illnesses, but rather a result of interactions between the gut and the brain. Many children with FAPDs frequently acquire further conditions associated with gut–brain interactions as they grow. Therefore, it is crucial to focus on adopting a healthy lifestyle and providing reassurance to both parents and children.

## 5 Study limitations

A limitation of this study is that the expert panel comprised exclusively pediatric gastroenterologists, without participation from primary healthcare providers. Although the expert panel provided comprehensive guidance, the inclusion of primary care practitioners might have added further practical insights relevant to first-line management. Additionally, the consensus-based methodology inherently includes an element of subjective expert judgment; however, this approach remains well-established and valuable, particularly in clinical areas where empirical data are limited. Lastly, although this expert panel represented diverse regions, variations in local healthcare resources and cultural practices may affect the universal applicability of some recommendations. Future studies incorporating direct patient or caregiver feedback, as well as broader involvement from primary care professionals, could complement these expert statements and further enhance their practical implementation.

## 6 Conclusion

In conclusion, diagnosing FAPDs poses challenges due to a lack of comprehension of the ROME IV diagnostic criteria among HCPs, particularly general practitioners and pediatricians. This condition, marked by chronic or recurrent abdominal pain without evident organic pathology, poses challenges in diagnosis and management. Addressing these knowledge gaps and increasing awareness are essential steps in improving the recognition and treatment of FAPDs in clinical practice. Clinical evaluation, excluding alarm features and identifying psychosocial factors, is essential. In the absence of organic issues, clinicians should contemplate the likelihood of a FAPD diagnosis. However, if there is suspicion of an organic disorder, clinicians must meticulously choose appropriate diagnostic tests. The expert panel recommends the implementation of probiotics as a part of a comprehensive approach along with incorporating dietary changes, psychological interventions, and other tailored therapies. Ongoing research on gut microbiota and emerging therapies, particularly probiotics, shows promise in addressing abdominal symptoms. While a holistic, multidisciplinary approach could be employed for optimal care of children with FAPDs, it may not always be feasible in primary care settings. This underscores the significance of developing accessible resources and guidelines to empower first-line HCPs with the knowledge and skills to effectively manage FAPDs.
