# Supplementary material for: Understanding functional abdominal pain disorders among children: a multidisciplinary expert consensus statement
Source: Front Pediatr. 2025 May 12;13:1576698. doi: 10.3389/fped.2025.1576698 (PMC12108103; doi:10.3389/fped.2025.1576698)
Supplement: Supplementary file 1 [file Table1.docx]

Supplementary Material

# Supplementary Table

Supplementary Table S1. Rome IV diagnostic criteria

| **Functional abdominal pain disorder** | **Diagnostic criteria** |
| --- | --- |
| **Irritable bowel syndrome** | The following criteria must be met for a minimum of 2 months:   - Abdominal pain along with changes in bowel movements, such as altered stool characteristics (stool frequency and appearance), occurs for a minimum of 4 days per month. - Abdominal pain persists even after constipation resolves. |
| **Functional dyspepsia** | The following pre-diagnostic criteria must be met for at least 2 months and must include one or more symptoms for at least 4 days per month:   - Postprandial fullness - Early satiety - Epigastric pain or burning sensation unrelated to bowel movements - After a thorough evaluation, the symptoms cannot be entirely attributed to another underlying medical condition. |
| **Abdominal migraine** | The following pre-diagnostic criteria must be met for a minimum of 6 months:   - Intermittent, severe, and prolonged periumbilical, midline, or diffuse abdominal pain lasting for at least 1 hour. - These episodes occur intermittently, with weeks to months between them, significantly impacting daily life activities and following a predictable pattern. - The pain is accompanied by two or more of the following: loss of appetite, nausea, vomiting, headache, sensitivity to light, or paleness. |
| **Unspecified functional abdominal pain** | The following pre-diagnostic criteria must be met for at least 2 months and four times per month.   - Intermittent or constant abdominal pain not linked solely to physiological events (e.g. eating or menstruation) - Does not meet the criteria for irritable bowel syndrome, functional dyspepsia, or abdominal migraine - After a thorough evaluation, the abdominal pain cannot be entirely attributed to another medical condition. |
